# Improved detection of disease-associated gut microbes using 16S sequence-based biomarkers

**DOI:** 10.1186/s12859-021-04427-7

**Published:** 2021-10-19

**Authors:** Brianna S. Chrisman, Kelley M. Paskov, Nate Stockham, Jae-Yoon Jung, Maya Varma, Peter Y. Washington, Christine Tataru, Shoko Iwai, Todd Z. DeSantis, Maude David, Dennis P. Wall

**Affiliations:** 1 Department of Bioengineering, Stanford University, Serra Mall, Stanford, USA; 2 Department of Biomedical Data Science, Stanford University, Serra Mall, Stanford, USA; 3 Department of Neuroscience, Stanford University, Serra Mall, Stanford, USA; 4 Department of Computer Science, Stanford University, Serra Mall, Stanford, USA; 5 Second Genome Inc, Allerton Ave, Brisbane, USA; 6 Department of Microbiology, Oregon State University, SW Campus Way, Corvallis, USA; 7 Department of Computer Science, Oregon State University, SW Campus Way, Corvallis, USA; 8 Department of Pediatrics (Systems Medicine), Stanford University, 1265 Welch Road, Stanford, USA

**Keywords:** 16S, Genomics, Microbiome

## Abstract

**Background:**

Sequencing partial 16S rRNA genes is a cost effective method for quantifying the microbial composition of an environment, such as the human gut. However, downstream analysis relies on binning reads into microbial groups by either considering each unique sequence as a different microbe, querying a database to get taxonomic labels from sequences, or clustering similar sequences together. However, these approaches do not fully capture evolutionary relationships between microbes, limiting the ability to identify differentially abundant groups of microbes between a diseased and control cohort. We present sequence-based biomarkers (SBBs), an aggregation method that groups and aggregates microbes using single variants and combinations of variants within their 16S sequences. We compare SBBs against other existing aggregation methods (OTU clustering and *Micropheno*or *DiTaxa* features) in several benchmarking tasks: biomarker discovery via permutation test, biomarker discovery via linear discriminant analysis, and phenotype prediction power. We demonstrate the SBBs perform on-par or better than the state-of-the-art methods in biomarker discovery and phenotype prediction.

**Results:**

On two independent datasets, SBBs identify differentially abundant groups of microbes with similar or higher statistical significance than existing methods in both a permutation-test-based analysis and using linear discriminant analysis effect size. . By grouping microbes by SBB, we can identify several differentially abundant microbial groups (FDR <.1) between children with autism and neurotypical controls in a set of 115 discordant siblings. *Porphyromonadaceae*, *Ruminococcaceae*, and an unnamed species of *Blastocystis* were significantly enriched in autism, while *Veillonellaceae* was significantly depleted. Likewise, aggregating microbes by SBB on a dataset of obese and lean twins, we find several significantly differentially abundant microbial groups (FDR<.1). We observed *Megasphaera* and*Sutterellaceae* highly enriched in obesity, and *Phocaeicola* significantly depleted. SBBs also perform on bar with or better than existing aggregation methods as features in a phenotype prediction model, predicting the autism phenotype with an ROC-AUC score of .64 and the obesity phenotype with an ROC-AUC score of .84.

**Conclusions:**

SBBs provide a powerful method for aggregating microbes to perform differential abundance analysis as well as phenotype prediction. Our source code can be freely downloaded from http://github.com/briannachrisman/16s_biomarkers.

## Background

The human gut microbiome contains as many bacterial, archael, and eukaryotic microbes as the number of human cells in the body [1]. These microbes perform metabolic functions, protect against pathogens, and engage in cross-talk with several human host organ systems, particularly with the immune system and the gut-brain axis [2–5]. With the advent of high-throughput next generation sequencing (NGS), researchers have turned to sequencing technologies to understand the characteristic microbial signatures of various human diseases.

**Fig. 1:**
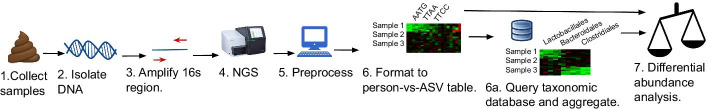
The steps of 16S sequencing (1–5) and traditional differential abundance analysis (6–7)

A common goal in such studies is to identify differentially abundant microbes between case and control clinical cohorts. 16S rRNA sequencing promises a cost-effective and computationally tractable way to achieve this. The 16S rRNA gene occurs in virtually all bacteria and archaea. It consists of highly conserved regions as well as 9 (V1–V9) hypervariable regions. These hypervariable regions (around 30–300 basepairs long) have evolved fairly vertically and have not changed rRNA function, thus serving as good phylogenetic markers. Microbial community profiling by 16S sequencing (Fig. 1) involves designing primers to target conserved sequences around a hypervariable region of choice, amplifying the region from a mixture of diverse genomes, and performing short read sequencing of the amplicons. These *exact sequencing variants* (ESVs) are then preprocessed to remove noise and sequencing artifacts in order *Amplicon Sequence Variants* (ASV) [6]. The final output of this pipeline is a matrix of read counts for each ASV.

Microbial groups to be tested for differential abundance are typically aggregated into groups in one of three ways (1) No aggregation is performed and each unique ASV is tested for differential abundance, (2) using classifiers (such as RDP, Kraken, METAXA, and Spingo) [7–10]) ESVs or ASVs are matched to a database containing known sequences and taxonomic classifications (Phylum, Class, Order, Family, Genus, Species, Strain), and aggregated over a chosen taxonomic level, or (3) similar sequences are aggregated together into operational taxonomic units (OTUs) (Fig. 1) or sequences with a certain biomarker are aggregated together. Each of these approaches has shortcomings, limiting the ability to identify differentially abundant microbes in a case versus control dataset.

### Individual ASVs

Using approach (1), high quality sequencing can identify several hundred distinct ASVs in an individual. Proponents of ASVs argue that low-error rate modern sequencing technologies can confidently resolve ASVs down to single nucleotide level and that this resolution may be important in identifying disease associated microbes [11–13]. However, ASVs can also be specific to household, region, or even sequencing batch [14], causing a matrix of relative abundance of person-vs-ASV to be very sparse and any disease-associated ASVs to have a small effect size. ASV matrices of the ocean microbiome samples, animal gut, raw milk cheese, and the human gut had sparsities of 90%, 79% 97% and 81%, respectively [15, 16]. To limit the number of multiple hypotheses being tested, researchers will often throw away ASVs that have low prevalence (the percent of samples that contain the ASV), even though they might have high abundance within a single sample and thus contains valuable information about an individuals’ microbiome composition [17]. Furthermore, is also possible for bacteria with different ASVs to be have identical functions in the context of the microbiome. An extreme case of this is when a single genome contains several different 16S operons [18], and can thus produces multiple different ASVs. Analyzing functionally identical bacteria as individual ASVs can weaken their apparent contribution in explaining host phenotype.

### Taxonomic category

Approach (2), relies on prokaryotic classification databases, such as the Ribosomal Database Project [19], GreenGenes [20], or Silva [21]. Although large-scale efforts are being made to expand such databases [22, 23] or develop taxonomic inference and imputation methods [24, 25], many databases only contain a fraction of the ASVs present in the human microbiome [26] and many taxonomic classifications are legacy namings that have not been updated with modern information about phylogenetic or functional relationships between microbes [27–29]. Additionally, taxonomy schema only have discrete levels (kingdom, phylum, class, order, family, species, and sometimes strain), not taking into account the continuous nature in which prokaryotes have evolved.

### OTU clusters and K-mer based groupings

Approaches of type (3) group ASVs or ESVs into aggregate groups either by overall sequence similarities (using discrete operational taxonomic units) or by grouping together ASVs or ESVs which contain various subsequences. Discrete operational taxonomic units (OTUs) are clustered together by sequence similarities, using a variety of hierarchical and Bayesian clustering methods [30–32]. This approach has the benefit over using individual read sequences in that clustering may get rid of artifacts in individual sequences caused by single nucleotide sequencing errors and batch effects. Clustering also does not rely on inherently incomplete databases. However, there is an ongoing debate regarding the best percent similarity cutoff to define OTUs (typically around 97%), and recent research suggests that the cutoff may differ depending on 16S region [33], and specific environment or disease being studied [11]. Moreover, OTU clustering algorithms tend to be extremely sensitive to small changes in hyperparameters or the structure of the data, and do not generalize well across different studies. [34–37]

Recently, grouping 16S sequence together by the presence of subsequences has emerged as an alternative to OTU clustering. Developed by the same group, *DiTaxa* [38] and *Micropheno* [39] are alignment-free approaches that group together microbes by the presence of different subsequences , with *Micropheno* generally having slightly higher performance than *DiTaxa* for most applications [38]. In both benchmarking studies, *Micropheno* outperformed OTU-clustering and *DiTaxa* in biomarker discovery power and host phenotype and environment prediction accuracy. *Micropheno* and *DiTaxa* are advantageous in that they are alignment-free and reference-free methods and do not require the computationally expensive alignment step. However, *Micropheno* does rely on shallow sub-sampling, using only a small subset of samples to a set of marker subsequences. While the authors showed that this subsampling sufficed in several different applications, tasks or environments with high sequencing error rates, high metagenomic diversity, or signals in low-abundance microbes may pose a challenge for shallow subsampling.

### Sequence-based biomarkers

**Fig. 2:**
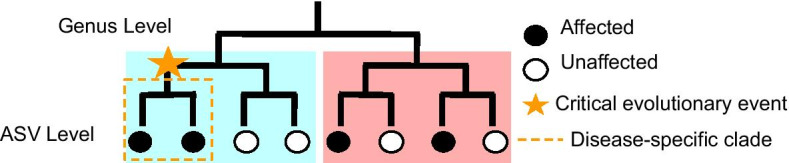
In this toy problem, ASVs are phylogenetically related by the given tree. Black circles correspond to ASVs found in the affected cohort and white circles correspond to ASVs in the control cohort. Although the there is a disease-specific clade enriched in the affected cohort, we would not necessarily detect this differential abundance using traditional analysis. On the ASV level, each ASV is only found in one individual, creating too sparse of a person-vs-ASV matrix to detect differential enrichment. Aggregating to the genus level, the affected and unaffected cohorts have similar abundance of genus 1 (blue) as well as genus 2 (red), and there is no genus-level differential enrichment

Since 16S sequences serve as evolutionary clocks, we hypothesize that we can group ASVs into clades by the presence of specific sequence-based biomarkers (SBBs) within the 16S sequence. Rather than using variants in 16S sequences to construct phylogenetic trees, which can be an inexact estimation of ancestry, computationally expensive, and sensitive to small changes in hyperparameters [40, 41], and then grouping ASVs by clades, we directly group ASVs together by polymorphisms in specific loci among the 16S region, implicitly aggregating 16S sequences from common ancestries. When it comes to aggregating microbes into groups, neither taxonomic categories nor OTUs or subsequence-based clustering are guaranteed to find the ’sweet spot’ between specificity and sensitivity (as shown in the toy example in Fig. 2). By testing for differential abundance over all possible SBBs, we eliminate the possibility that we miss a differentially abundant group of microbes by not considering the appropriate aggregation level.

In this study, we show that SBBs perform on par with or outperform OTU clustering,*Micropheno*, and *DiTaxa*, two other other state-of-the art taxonomy-free aggregation methods. We use three different benchmarking tasks: (1) differential abundance using an in-house permutation-based pipeline, (2) biomarker discovery using *LefSeq*, a popular linear-determinant-analysis based method [42], and (3) phenotype prediction using random forest classifiers. We analyze the performance of the grouping strategies on two different gut microbiome datasets: a dataset of obese and lean twins and a dataset of children with autism and their neurotypical siblings.

## Results and discussion

### Multi-loci SBBs yield high statistical power in identifying differentially abundant microbial groups

**Fig. 3:**
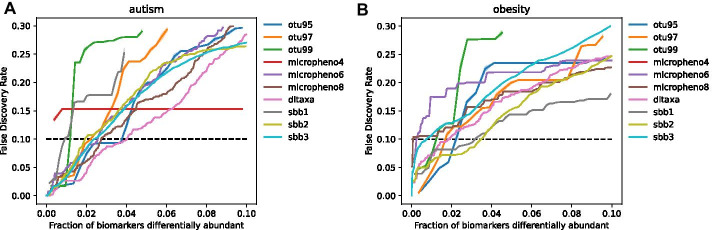
Biomarker discovery power for different aggregation strategies using a permutation-based test. The dotted line signifies a false discovery rate of .1 **a** FDR curves for biomarker discovery for microbial groups differentially enriched in autism. **b** FDR curves for biomarker discovery for microbial groups differentially enriched in obesity

Using sequence-based biomarkers, we were able to identify groups of microbes differentially enriched in autism as well as in obesity with high statistical power. From comparison between the true and simulated null distributions of differential enrichment test statistics, we computed fraction of significant microbial groups versus false discovery rate (FDR). We show this in Fig. 3 for each microbial group, aggregated by different aggregation methods. We note several important observations.

First, for autism and obesity, SBBs provide high discovery power in identifying differentially enriched groups of microbes (Fig. 3). In using 2- and 3-loci biomarkers under a false discovery rate of 0.1, we find 2.5% of microbial significant in autism, and in using 1- and 2-loci biomarkers, 3.5% of microbial groups were significant in obesity. Note that evaluating discovery power based on percent of aggregated groups, rather than number of aggregated groups, is a more appropriate comparison because multi-loci SBBs have many more possible microbial groupings, and will inherently have larger number of significant microbial groups than other aggregation methods. In the autism dataset, many grouping methods performed similarly well, including 2-loci and 3-loci SBBS, and in the obesity datset, 1- and 2-loci SBBs outperformed all other aggregation methods, identifying nearly twice as many significant groupings as the next best aggregation strategy.

Using *LefSeq* to identify biomarkers with differential abundance between case and control, we found that SBBs outperformed OTU clustering in most comparisons (Tables 1, 2). In both datasets, 2-loci SBBs outperformed OTU clustering and micropheno in terms of median effect size, and in the obesity dataset, 1- 2-loci SBBs had a similarly high significant fraction of biomarkers as the best performing method.

**Table 1:** *LefSe* results for the autism/neurotypical dataset computed for different types of biomarkers. In bold are the highest performing results, and results within a standard deviation, for fraction differentially abundant microbes and median effect size

Differential biomarkers in autism identified using *LefSe*		
Biomarker type	Fraction differentially abundant	Median effect size
otu95	0.168	0.009
otu97	0.161	0.017
otu99	0.128	0.020
micropheno4	**0.237**	0.021
micropheno6	0.149	0.014
micropheno8	0.112	0.017
ditaxa	0.129	0.017
sbb1	0.158	0.018
sbb2	0.112	**0.023**
sbb3	0.098	0.012

**Table 2:** *LefSe* results for the lean/obesity dataset computed for different types of biomarkers. In bold are the highest performing results, and results within a standard deviation, for fraction differentially abundant microbes and median effect size

Differential biomarkers in obesity identified using *LefSe*		
Biomarker type	Fraction differentially abundant	Median effect size
otu95	0.292	0.008
otu97	0.275	0.011
otu99	0.239	0.012
micropheno4	0.222	0.013
micropheno6	0.312	0.008
micropheno8	**0.331**	0.008
ditaxa	**0.356**	0.009
sbb1	**0.329**	0.004
sbb2	**0.323**	0.013
sbb3	0.260	**0.020**

Finally, we note that although the autism and obesity datasets used different variable regions of the 16S sequence (V4 and V3), and were sequenced at different facilities using different sequencing pipelines, SBBs provided a powerful method for differential enrichment analysis for both datasets.

### Multi-loci SBBs can be used as features for phenotype prediction models

**Fig. 4:**
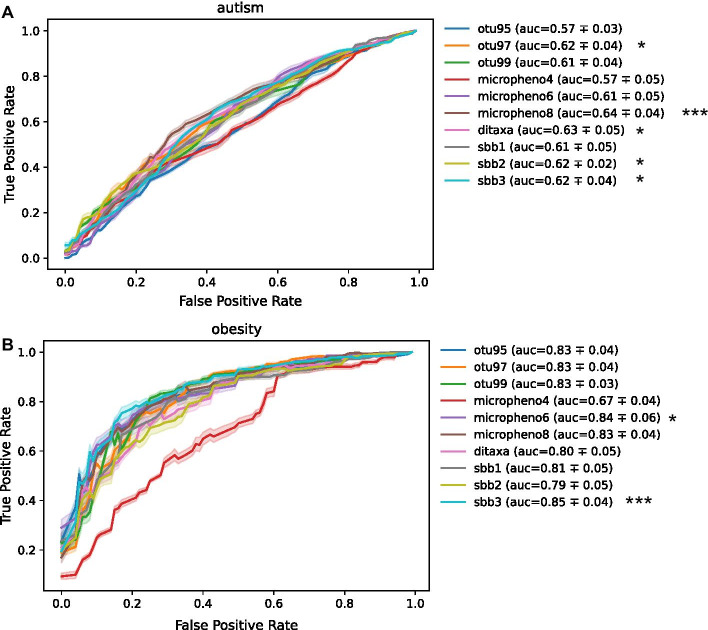
ROC curves and ROC-AUC scores for phenotype prediction using random forest classifiers and different aggregation strategy groupings as features. Both ROC and ROC-AUC confidence intervals were using a 80%/20% train-test split for 100 iterations. **a** ROC curves for prediction of the autism phenotype using various types of aggregation methods to extract features **b** ROC curves for prediction of the obesity phenotype using various types of aggregation methods to extract features

Using a random forest classifier, we built host phenotype prediction models using the aggregate abundances output by different aggregation methods as features. Several studies have shown that random forests have high performance in predicting host phenotype from microbiome data [39, 43–45], and random forests have a high interpretabilty, ease-of-use, and work robustly with many different types of feature sets. As shown in Fig. 4, SBBs perform on par with the state-of-the-art aggregation methods, with 3-loci SBBs outperforming all methods in the obesity dataset.

### 2-loci SBBs provide good balance between computation time and microbial group specificity

While different hyperparameter setting for each aggregation strategy had varying performance, 2-SBBs performed on par with or higher than all other aggregation strategies on all tasks for both datasets. We hypothesize that for both the V3 region used in the obesity dataset, and the V4 region used in the autism dataset 2-loci mutations are enough information to group microbes together in a biologically relevant way. However, we note that the, the size of the region, hypermutability of 16S region, and number of ASVs in a dataset could influence which order of SBB is appropriate. Related, several analyses have shown that the choice of 16S region influences the taxonomic level that OTU clustering-by-similarity corresponds to [36, 46]. Additionally, 2-loci SBBs. However, the pipeline for 3-loci and higher order SBBs is much more computationally expensive since there are several hundred-fold more 3-loci SBBs to extract from sequences, and with which to compute abundance and perform a significance testing. Given the computational cost of higher order SBBs, and the advantage of not having to do hyperparameter tuning, we propose that 2-loci SBBs as the standard selection for SBB feature extraction.

### SBBs implicate known disease-associated microbes in autism and obesity

**Fig. 5:**
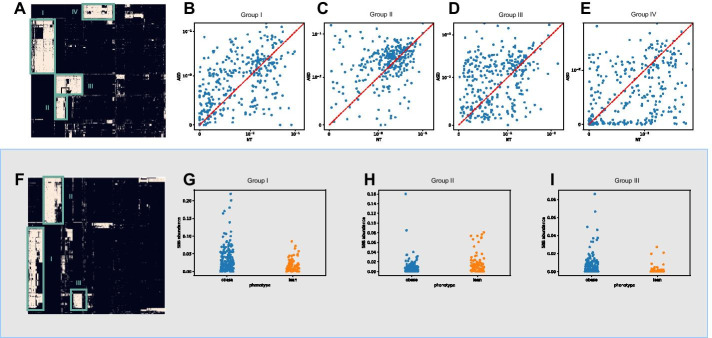
**a** Hierarchical clustering of the most significant FDR<.1) biomarkers vs the ASVs that contain them identifies several distinct clusters of microbes differentially enriched in autism. **b**–**e** An example of differential enrichment of a randomly chosen biomarker from within each group: **b** Group 1 consists of primarily of *Ruminococcaceae*, enriched in children with autism. **c** Also enriched in autism, group 2 consists of many members of the *Porphyromonadaceae* family. **d** Group 3 consists of primarily of unclassified *Blastocystis*. **e** Group 4 consists of consists of primarily members from the *Veillonellaceae* family, and is underenriched in autism. **f** Hierarchical clustering of the most significant (FDR<.1) biomarkers vs the ASVs that contain them identifies several distinct clusters of microbes differentially enriched in obesity. Although the SBBs differentially enriched in obesity do not cluster into as discrete groups of ASVs as in the ASD study, we point out several possible clusters. **g**–**I** An example of differential abundance of a randomly chosen biomarker from within each group. **g** Group 1 consists of primarily *Megasphaera*, enriched in the obese cohort. **h** Group 2 consists of primarily *Phocaeicola*, enriched in the obese cohort. **i** Group 3 consists of primarily *Sutterellaceae*, enriched in the lean cohort

Although we pruned biomarkers in complete LD, multiple biomarkers can still correspond to similar sets of ASVs. For each dataset, we performed hierarchical clustering (using a hamming distance) on the binary matrix of ASV-vs-biomarkers to identify conserved sets of bacteria that contain many of the significant SBBs. From Fig. 5, we highlight several conserved groups of bacteria. We then query these sequences from a taxonomic database for validation.

In autism, groups consisting primarily *Ruminococcaceae* and *Porphyromonadaceae* families were enriched in the autism cohort, and *Veillonellaceae* underenriched in the autism cohort. This is in agreement with a previous studies [47], which found *Ruminococcaceae* enriched in children with autism and gastrointestinal disturbances, and *Veillonellaceae* underenriched in children with autism [48]. *Porphyromonadaceae* has been linked to major depressive disorder, suggesting a role for it in the gut-brain axis [49].

Using SBBs, we found groups consisting of *Megasphaera* and *Sutterellaceae* enriched and groups consisting primarily of *Phocaeicola* and under-enriched in obesity. The original authors of the obesity dataset [50] also found *Megasphaera* to be enriched, using taxonomic aggregation methods. Other studies have found that *Sutterellaceae* is enriched in obesity [51], and *Phoecaeicola* fluctuates with diet [52]. Thus, SBB groupings capture similar biological relationships as taxonomic aggregation or OTU clustering, without the burdens of relying on an external database or algorithms highly dependent on hyperparameters and datasets.

### SBBs implicate *Blastocystis*, a phylogenetically challenging microbe, in autism

Despite the strong differential abundance and prevalence between children with autism and controls observed in our study, *Blastocystis* has not previously been implicated in autism or neurodevelopmental conditions. Although *Blastocystis* is a protozoan rather than a prokaryote, it contains an 18S rRNA that is amplified by 16S sequencing primers [53]. Consequently, *Blastocystis* 18S sequences are very distant from prokaryotic 16S sequences, and may be thrown out in microbial analysis in an attempt to reduce non-16S contamination [54]. Furthermore, reads from *Blastocystis* are challenging to aggregate taxonomically: *Blastocystis* does not exist in the GreenGenes or Silva 16S databases (in our annotation pipeline, the most specific taxonomic annotation for such a sequence was the bacteria kingdom, which is not even correct; NCBI BLAST verified these sequences belonged to *Blastocystis*) so cannot be aggregated by taxonomy; likewise subtypes of *Blastocystis* are not genetically similar enough to each other to be put in the same group via OTU clustering with the standard similarity cutoffs ( 97% sequence similarity). However, using SBBs we clearly identified conserved bases in the amplified region of *Blastocystis* that allowed us to group them together concretely enough to identify a group of differentially enriched *Blastocystis* in autism.

*Blastocystis* are a physiologically intriguing microbe. Contradicting studies have identified *Blastocystis* as commensal microbes in the healthy human gut and as as dangerous pathogens [55, 56]. Interestingly, one hypothesis proposed that increased *Blastocystis* infections arising from increased travel, interaction with livestock, and sewage pollution, may be a primary cause for the rise in autism incidence in Europe and North America [57]. It should be noted that this hypothesis is purely speculative, and even from our results, the direction of causality is unclear. However, given the strong differential prevalence and abundance of *Blastocystis* in autism in our results, future autism microbiome studies should consider including *Blastocystis* in their analysis.

## Conclusions

We have demonstrated that using sequence-based biomarkers to group together 16S sequences is a powerful aggregation method. Grouping microbes by SBBs involves very little hyperparameter tuning and does not rely on reference databases. In addition to identifying differentially enriched microbial groups with low false discovery rates, SBBs aggregate 16S sequences into biologically relevant groups that both capture the same differentially enriched microbes as traditional aggregation methods, but also into novel groups of microbes with high statistical significance and biological relevance.

While we conclude that SBBs are a powerful aggregation method, we note that they cannot infer causal microbiome compositions linked to either autism or obesity. However, we hope that our preliminary results on differential microbial enrichment, especially in autism, will pave the way for future analyses on the relationship between the gut microbiome and complex disease.

The success of our method has several additional implications for microbiome analyses. First, we have shown that 16S sequencing can yield powerful results. 16S sequencing is affordable and computationally easy to work with compared to whole metagenomic sequencing. Although 16S sequencing has been criticized for not yielding valuable insights, and many studies jump directly in metagenomic sequencing, we illustrate that preliminary analysis can and should be done on 16S sequencing before spending time and resources on metagenomics. Additionally, despite the growing popularity of shotgun metagenomics, the generation of raw 16S data continues to outpace shotgun metagenomics, making 16S data a valuable data type for microbiome research. According to Pubmed, in 2019 170 articles with associated 16S microbiome datasets were published, compared to 54 articles with associated metagenomics datasets [58].

Additionally, a common step in many gut microbiome preprocessing pipelines is to discard the least prevalent ASVs. Prevalence is defined as the mean number of samples (persons) in which an ASV was present. However, even if an ASV has a low *prevalence*, it may still be very *abundant* in the samples that it is present in, meaning that it yields important information about gut microbiome on a sample level. Our method does not discard these valuable data, and is able to still gain insight from low prevalence, high abundance ASVs, as they get aggregated over biomarkers. Finally, the only parameters used in our method were the choice of MSA algorithm (for which our choice, the aligner from the RDP set of tools [19], yielded powerful results) and the number of variant combinations that went into the biomarker. This further highlights the power and robustness of this method. Our results using SBBs pave a new avenue for harnessing the power of 16S sequencing to understand microbial signatures of complex human disease.

## Methods

### Data collection and preprocessing

#### Paired autism and neurotypical siblings

Stool samples were collected at home from 115 pairs of children with autism and their neurotypical siblings. Stool biospecimens were placed in transfer buffer and shipped at ambient temperature then placed in freezer upon arrival at Second Genome (Brisbane, CA) in all cases within 3 days of collection. DNA was extracted with the PowerMag$\circledR$ Microbiome RNA/DNA Isolation kit (Qiagen, Carlsbad, CA) and the ZR-96 DNA Clean-up kit (Zymo, Irvine, CA). The V4 variable region of the 16S rRNA gene sequence was PCR-amplified using fusion primers designed against the conserved regions and tailed with Illumina adapters and indexing barcodes, and sequenced using the MiSeq$\circledR$ for 250 cycles as described in (citation XYX). Raw sequence reads were processed with DADA2 applying default settings for filtering, learning errors, dereplication, ASV inference, and chimera removal [59].

#### Obese lean twins dataset

We apply our methodology to a published dataset that sought to identify differentially abundant microbes in obese patients [50]. This landmark study used 16S sequencing targeting the V3 region of 196 obese and 61 lean individuals. We accessed the pre-processed fastq files via QIITA, an online database of public microbiome studies [60, 61].

### Normalization

In order to test if a particular ASV or group of ASVs is enriched in a diseased cohort, we use the following procedure.

Normalize read count of each ASV *a* in person *p*, ${\mathbf {C}}_{a,p}$ to relative abundance ${\mathbf {R}}_{a,p}$ using Eq. 1:

1 $$\begin{aligned} {\mathbf {R}}_{a,p}= & {} \frac{{\mathbf {C}}_{a,p}}{\sum \limits _{a} {\mathbf {C}}_{a,p}} \end{aligned}$$

2 $$\begin{aligned} \mathbf {R'}_{G,p}= & {} \sum \limits _{a \in G} {\mathbf {R}}_{a,p} \end{aligned}$$

Relative abundance is a commonly used normalization procedure in microbiome studies. Because 16S count data is compositional, normalizing by relative abundance might invalidate certain downstream analyses, such as measurements of alpha and beta diversity, using statistical metrics that assume equal variance or errors across groups, and multiple hypotheses corrections that assume independence between relative abundances of different microbes [62, 63]. However, our downstream conservative testing procedure relies on non-parametric statistics and permutation tests, and any possible biases or inaccuracies using normalization will be thus accounted for in the permuted null distribution [64].

### General aggregation strategy

If aggregating ASVs into groups, for person *p* and group *G* , we sum the relative abundance of each ASV belonging to group *G* in order to get the total relative abundance of each group for each person. For *Micropheno*-grouped biomarkers, we tested k-mers of length 4, 6, and 8. For *DiTaxa*-grouped biomarkers, we used the default settings: a corpus size of 50,000 subsequences with no subsampling.

### Aggregating by sequence-based biomarker

In order to aggregate 16S sequences into groups by SBBs, we perform a multi-step algorithm where we (1) perform multiple sequence alignment, (2) compute the presence of each SBB in each ASV, (3) remove SBBs in linkage disequilibrium, and (4) aggregate relative abundance of microbes across each SBB.

First (1), we perform multiple sequence alignment using all the sequences in a dataset. We chose RDP’s 16S alignment tool to perform MSA on the 16S sequences. RDP’s alignment tool, one of the most commonly used alignment methods, uses an infernal aligner that uses covariance models to align structurally similar RNA sequences, such as the 16S region [65].

Next (2), we compute the presence of each sequence-based biomarker in 16S sequence. For single-loci variants, we use the presence of a base (*A*, *T*, *C*, *G*) at a position along the aligned sequence as a biomarker. If an ASV contained an *A* at (post-MSA) position 1, *C* at position 2, and *G* at position 3 then it would contain the biomarkers *1A*, *2C*, and *3G*. We hypothesized that it was possible single-loci biomarkers would not provide enough granularity (approximately a quarter of microbes should contain a given base at each position, and thus will get grouped together). Thus, we created biomarkers from combinations of single variants, with the aim of achieving smaller and more granular groupings of bacteria. We tried combinations of 2 and 3 variants. ASV containing *A* at position 1, *C* at position 2, and *G* at position 3 would contain the biomarkers *1A/2C*, *1A/2C*, and *2C/2G* (if looking at combinations of 2 loci) and *1A/2C/3G* (if looking at combinations of 3 loci).

**Fig. 6:**
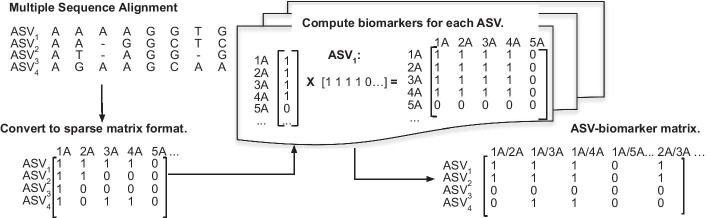
Extracting an ASV-biomarker matrix can be visualized as a sparse-matrix multiplication problem: Starting from multiple sequence alignment, we format the MSA into a one-hot-encoding representation of ASVs versus variants. For each ASV, we compute the presence of multi-loci biomarkers by taking the square product (for 2-loci biomarkers), or cube (for 3-loci biomarkers) of the ASV’s row in the ASV-variant matrix in order to compute a ASV-vs-biomarker sparse matrix $\mathbf{B}$. We can then multiply this with person-vs-ASV relative abundance matrix $\mathbf{R}$ to compute relative abundance matrix of person-vs-biomarker $\mathbf{R}'$

The output of this step is a binary $n_a$ by $n_b$ matrix, ${\mathbf {B}}^N$ where $n_a$ is the number of ASVs and $n_b$ is the number of total possible biomarkers across all ASVs. ${\mathbf {B}}^{N}_{a,b}$ corresponds to whether or not ASV *a* contains *N*-loci-combination biomarker *b*. We show the workflow of this step in Fig. 6.

It is possible for biomarkers to be in complete linkage disequilibrium (LD) with each other and thus contain redundant information. For example, if every 16S sequence that has the biomarker 1*C*/2*A* also contains the biomarker 5*C*/8*G*, then the two biomarkers would be in LD. In step (3), we perform LD-pruning on the set of biomarkers before performing differential abundance testing.

Finally (4), we compute relative abundance of each SBB by aggregating the relative abundance of sequences containing the SBB, similar to how we aggregated across taxonomic category.

### Biomarker discovery

#### Permutation-based false discovery rate

After aggregating across ASV (either by SBB or taxonomic ranking as described above), for each microbial group, we derive an FDR using a non-parametric statistical tests comparing the distributions of the case and control groups.

For paired data, such as in the autism-neurotypical sibling study we compute test statistics using the Wilcoxon rank sum test, a non-parametric test for paired data. For unpaired data, such as the lean and obese dataset (although the dataset contains twins, they are concordant for the obesity phenotype), we compute a test statistic using the Mann-U-Whitney test, a non-parametric test for unpaired data. Computing these test statistics for each microbial group is an embarrassingly parallel operation and we parallelize this across multiple cores.

We used a permutation test to compute false discovery rate to determine test statistic cutoff for multiple hypothesis correction. Since for 2-loci and 3-loci SBBs, there can be over 100,000 possible microbial groups to test differential abundance for, a Bonferonni correction limits the capacity to detect true positives. Moreover, because an ASV can fall into multiple groupings using SBBs, and individual ASVs may even be correlated, a permutation test allows us to control the family-wise error rate with a null distribution that simulates the non-independence between microbial groups. Additionally, a null distribution via permutation test allows us to control for the relationship between related individuals in either study. We permuted each dataset as follows: for each iteration, we shuffled the case and control labels for the dataset. For the autism dataset, for each permutation we either changed both or neither of the phenotypes for each case-control pair. For the obesity dataset, we kept phenotype concordance across siblings constant, as well as the number of case and control labels. We performed 100,000 iterations, and computed the FDR for differential abundance of each microbe or group, and used this to simulate a null distribution and subsequently control FDR.

#### Biomarker discovery via linear determinant analysis effect size (*LefSe*)

Using the sample-vs-abundance matrix of each type of aggregation strategy,we used the standard *LefSe* [42] pipeline to identify biomarkers with significantly different abundances between case and control. Because *LefSe* is not optimized to run on hundreds of thousands of features, which is the order of magnitude of the SBB-2 and SBB-3 matrices, we subset each feature matrix to the top 1000 features with the highest variance. The reduced the number of features tested for both the SBB-aggregated matrices and the *Micropheno*-aggregated and *DiTaxa*-aggregated matrices. We used the default parameters for the *LefSe* to build the *LefSe* input data and to perform linear determinant effect size analysis.

### Phenotype prediction model

To predict phenotype from 16S features, we built random forest classifier using scikit-learn. We used the default parameters and 100 forests. To compute confidence intervals for our ROC curves and AUC scores, we performed the following pipeline 100 times: We split each dataset into 80% training data and %20 testing data, trained the random forest classifier on the training dataset and then predicted on the test dataset. From the output prediction probabilities we computed false and true positive rates, and the value for ROC-AUC value. We then used the set of 100 values to compute the medians and confidence intervals (1 standard deviation out) for the ROC curve and the AUC scores.

## Data Availability

Raw data for the obesity dataset can be accessed from the QIITA database [60] under ID 77 [61], and can be found at https://qiita.ucsd.edu/public/?artifact_id=6821. ASV tables, sample metadata, and annotated ASVs for both the autism dataset and obesity dataset can be accessed at https://github.com/briannachrisman/16s_biomarkers/tree/main/public_data. Source code for the SBBs methodology is freely available at https://github.com/briannachrisman/16s_biomarkers.

## Abbreviations

SBB: sequence-based biomarker
MSA: multiple sequence alignment
NGS: next-generation sequencing
ASV: amplicon sequencing variant
OTU: operational taxonomic unit
LD: linkage disequilibrium
